# Clinico-Radiological Correlation Between Anterior Cruciate Ligament Deficiency and Hyperextension of the Knee Joint: A Prospective Study

**DOI:** 10.7759/cureus.59817

**Published:** 2024-05-07

**Authors:** Mansingh Jarolia, Hira L Nag, Sai Krishna MLV, Shivanand Gamanagatti

**Affiliations:** 1 Orthopedics, All India Institute of Medical Sciences, New Delhi, New Delhi, IND; 2 Radiology, All India Institute of Medical Sciences, New Delhi, New Delhi, IND

**Keywords:** acl tear.‎, posterior tibial slope., notch width index, hyperlaxity, knee hyperextension

## Abstract

Introduction: The anterior cruciate ligament (ACL) primarily restricts anterior sliding of the tibia over the fixed femur, thereby also postulating to prevent hyperextension of the knee joint. The main objective of our study was to identify the role of the ACL in the prevention of knee hyperextension and to quantify the amount of hyperextension caused by an ACL tear, apart from its well-established role in the prevention of anterior tibial translation on the fixed femur.

Methods: This prospective study was conducted in a tertiary care hospital. Eighty patients with unilateral ACL tears were assessed clinico-radiologically in the preoperative period to quantify the knee hyperextension, which was then compared with the uninjured contralateral knee of the same patient. Posterior tibial slope and notch width index were also assessed to rule out bias in our study.

Results: The mean age of patients in our study was 27.3 years. Out of 80 patients, 70 were male and 10 were female. The Pearson coefficient for clinically and radiologically assessed hyperextension was 0.919 (p-value 0.001) and 0.910 (p-value 0.001), respectively. Posterior tibial slope and notch width index assessment showed Pearson coefficients of -0.018 (p-value 0.887) and -0.068 (p-value 0.547), respectively.

Conclusion: Anterior cruciate ligament complete tear or deficiency produces knee hyperextension, which varies from patient to patient. Though the amount of hyperextension produced is mild (less than five degrees in most patients), it can cause a significant amount of knee instability. Hence, correction of knee hyperextension is crucial while performing ACL reconstruction.

## Introduction

Anterior cruciate ligament (ACL) injury is the most common ligamentous injury in the knee joint. Men have a significantly higher annual incidence compared to women [1]. Female athletes have been identified as having an increased risk of ACL injury during same-level sports activities compared to male athletes [2,3]. There is a remarkable escalation in arthroscopic ACL reconstruction surgeries to primarily treat ligamentous instability, secondarily to prevent further degenerative changes in the knee joint, and also to facilitate early rehabilitation and return to preinjury levels of physical activity. The percentage of ACL reconstructions performed in an outpatient setting increased from 43% to 95% between 1994 and 2006 and is still on the rise [4].

The ACL primarily restricts anterior sliding of the tibia over the fixed femur, thereby also postulating to prevent hyperextension of the knee joint. Knee hyperextension is operationally defined as more than a 05° extension of the knee joint [5].

The posterior structures of the knee are likely to be stressed in an individual who displays knee joint hyperextension. Deficiency of these soft tissues may have a significant role in knee hyperextension. A cadaveric study on fresh frozen knees demonstrated maximum knee hyperextension with sectioning of the oblique popliteal ligament, independent of cutting order, followed by the anterior cruciate ligament, while the least contribution was offered by the fabello-fibular ligament and posterior tibial slope [6].

The knee travels farther in its final arc and produces higher anterior translatory forces when there is pre-existing excessive hyperextension. Hence, an increased frequency of ACL rupture might result from hyperextension of the knee. The literature regarding the same is still ambiguous. Borsa et al. showed that the greatest anterior translation forces are seen when the quadriceps are activated at higher acceleration with small knee flexion angles [7]. Terauchi et al. found that there was no significant difference in the mean extension angle between ACL-deficient groups and controls when measured on MRI [8].

The present study aimed to identify the relationship between ACL deficiency and knee hyperextension, considering the uninjured contralateral knee as a control to compare knee hyperextension with the ACL-deficient knee.

## Materials and methods

Study design

This is a cross-sectional study carried out to evaluate hyperextension with the help of clinical examination and radiological examination in ACL-injured patients.

Ethical considerations

Due ethical clearance was obtained from the Institute Ethical Clearance (IECPG-442/29.11.2017, RT-2/20,12.2017).

Study criteria

Eighty patients with unilateral isolated ACL injuries with contralateral normal knee joints were recruited. Patients with ages less than 18 years and more than 55 years were excluded. Patients with multi-ligamentous injuries to the ipsilateral knee and any surgical procedure done previously in either of the knees were excluded from the study.

Sample size calculation

Considering the prevalence of hyperextension (28%), with 10% absolute precision and a 95% level of confidence interval, we recruited 80 ACL-deficient patients after fulfilling inclusion and exclusion criteria.

Procedure 

Preoperatively, all patients were examined clinically and radiologically to assess knee hyperextension. Anterior drawer test, Lachman test, and pivot shift tests were performed as a part of a routine clinical examination of ACL tear, which was confirmed by an MRI of the knee joint. All included patients were clinically evaluated to look for associated signs of generalized ligamentous laxity using the Beighton score to rule out other causes of knee joint hyperextension [9].

Clinical assessment of hyperextension of knee joint

The examiner stabilized the distal femur above the epicondyles with one hand while the other hand applied an anteriorly elevating force to the leg by lifting the great toe. This maneuver was performed on both the injured and uninjured contralateral knees, and any asymmetry, if present, was measured [10].

**Figure 1:**
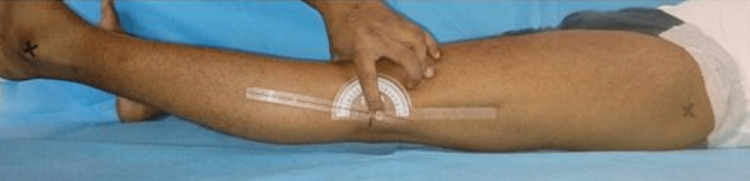
Clinical measurement of knee hyperextension. The goniometer is placed at the lateral knee joint line; one limb of the goniometer directed toward the lateral malleolus, and the other limb is directed toward the greater trochanter.

**Figure 2:**
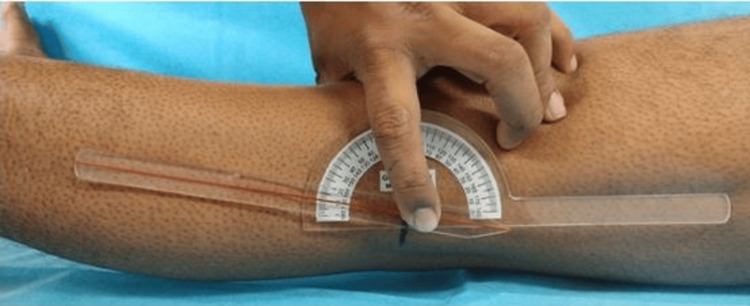
Showing the amount of knee hyperextension measured on the left knee joint of the patient.

The patient was kept supine on the examination couch, and then the tip of the lateral malleolus, knee joint line, and greater trochanter were marked. One limb of the goniometer was applied to the lateral thigh in line with the femur directing to the greater trochanter, while another limb of the goniometer was applied to the leg directed to the lateral malleolus with the center of the device over the knee joint (Figure 1). The distal hand hyperextended the knee by raising the leg from the heel, while the proximal hand, which was holding the goniometer, securely kept the thigh down to the bed so that the popliteal fossa was in touch with the couch (Figure 2). This led us to measure clinical knee joint hyperextension. Clinical measurement of knee hyperextension was done by one orthopedician and two physiotherapists. The mean of all three measurements was calculated and used as a final value to minimize bias and inter-observer variation. 

Radiological assessment of knee joint hyperextension

**Figure 3:**
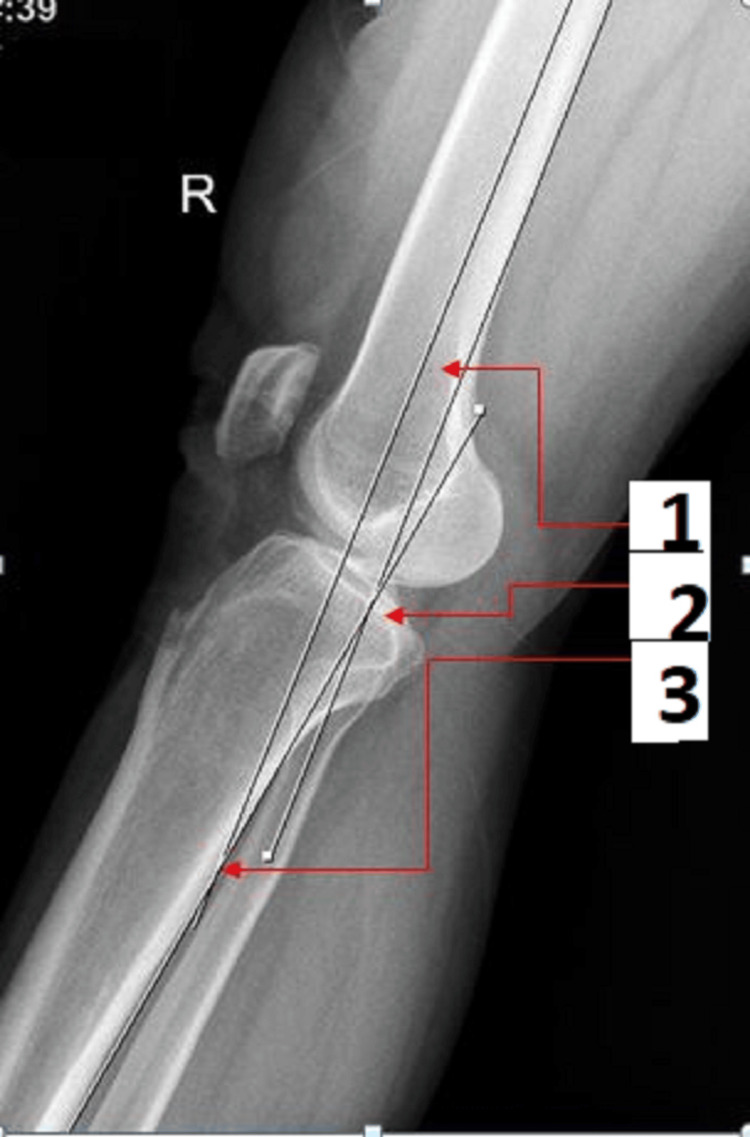
True lateral view X-ray of the knee taken in hyperextension. One line was drawn parallel to the posterior cortex of the femur, and the second line was drawn parallel to the posterior cortex of the tibia. The angle of hyperextension was measured at the point where two lines were transecting each other. 1- A line parallel to the posterior cortex of the femur. 2- Hyperextension angle. 3- A line along the posterior cortex of the tibia.

The popliteal fossa was hung 10 cm above the X-ray table with the patient positioned supine on it and the heel resting on a foam bolster. The patient was told to let gravity fully extend their knee by relaxing their leg. The knee was in the center of a cassette. The X-ray beam was parallel to the joint line and aimed medially and laterally. Until the lateral projections of the medial and lateral condyles were superimposed, roentgenograms were performed. Films with a medial or lateral femoral condyle offset of 5 mm or less were used for measurement. The angle created by the junction of lines drawn parallel to the posterior cortex of the femur and tibia was used to calculate knee extension (Figure 3) [11]. Radiological measurement of knee hyperextension was done by one orthopedician and two radiologists; the mean of all three measurements was calculated and used as a final value to minimize bias and inter-observer variation.

Statistical analysis

Data was recorded and analyzed using IBM Corp. Released 2010. IBM SPSS Statistics for Windows, Version 19.0. Armonk, NY: IBM Corp. Results from continuous measurements were presented as Mean. Significance was assessed at a 5% level of significance. To evaluate the correlation for parametric data, the Pearson product-moment correlation coefficient (r) has been used (±1 perfect correlation and 0 no correlation).

## Results

A total of 80 patients were evaluated, ranging in age from 18 to 55 years old (Table 1). The mean age was 27.3 (+/- 8.2) years. Seventy patients were male, and 10 were female (Table 2). The commonest mode of injury was road traffic accidents (Table 3). 

**Table 1:** Age distribution of ACL injured patients.

Range of age	Number of patients
18-25 years	42
26-35 years	22
>35 years	16
Total patients	80

**Table 2:** Sex distribution of patients.

Gender	Number of patients
Male	70
Female	10

**Table 3:** Mode of injury of ACL injured patients.

Mode of injury	Number of patients
Sports injury	32
Road traffic accidents /other	48
Total	80

The clinically mean degree of hyperextension in patients without general ligamentous laxity in the affected knee was 8.57° compared to 8.03° of the contralateral uninjured knee joint, while patients with general ligamentous laxity had 11.42° of knee hyperextension compared to 10.42° of knee hyperextension of the contralateral joint. The radiologically measured mean degree of hyperextension in patients without general ligamentous laxity in the affected knee was 8.60° compared to 7.68° of the contralateral knee joint, while patients with general ligamentous laxity had 11.20° of knee hyperextension compared to 10.38° of knee hyperextension of the contralateral joint.

The maximum degree of hyperextension measured in the affected knee was 20° compared to 18° of the contralateral knee joint of the same patient, and the minimum degree of hyperextension measured in the affected knee was 5° compared to zero degrees of the contralateral knee joint of the same patient. The maximum amount of knee hyperextension difference between the ACL-deficient knee (13.70°) and the normal knee (6.33°) was 7.37°, while the minimum amount of knee hyperextension difference was 0°.

**Figure 4:**
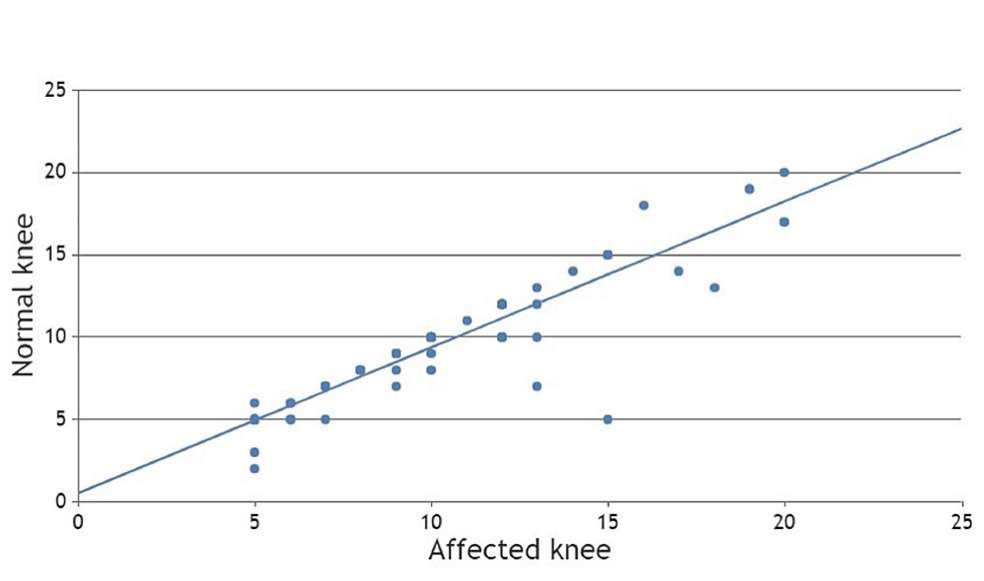
Scatter chart of clinical hyperextension between the affected knee and the normal knee.

Clinically evaluated knee hyperextensions of the injured knee and uninjured contralateral knee were analyzed using the Pearson correlation coefficient method (Figure 4). The Pearson correlation coefficient was 0.919, which suggests a strong positive correlation between hyperextension of the knee joint and ACL tears. The p-value was 0.001, which suggests there was a linear correlation between ACL tear and hyperextension of the knee joint (Table 4). 

**Table 4:** Statistical analysis of clinically assessed hyperextension of the knee joint.

Clinical Parameters		Hyperextension (Affected Knee)	Hyperextension (Normal Knee)
Hyperextension (Affected knee)	Pearson Correlation	1	0.919
	Sig. (2-tailed)	-	0.001
	N	80	80
Hyperextension (Normal knee)	Pearson Correlation	0.919	1
	Sig. (2-tailed)	0.001	-
	N	80	80

**Figure 5:**
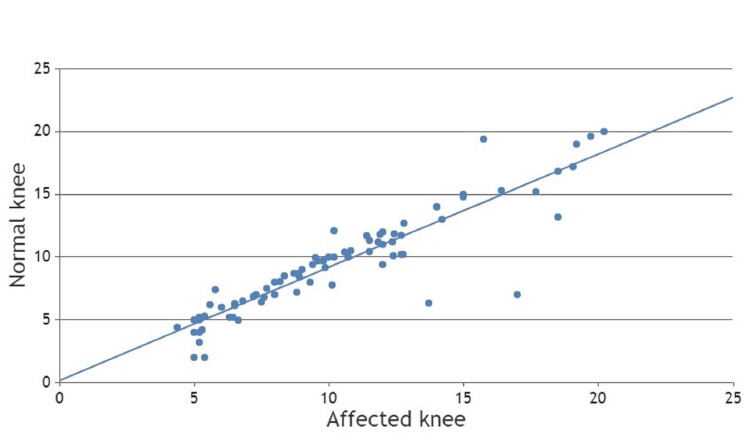
Scatter chart of radiological hyperextension between the affected knee and the normal knee.

Similarly, radiologically evaluated knee hyperextension was also assessed using the Pearson correlation method (Figure 5). The Pearson correlation coefficient was 0.910, which suggests a strong positive correlation between hyperextension of the knee joint and ACL tears. The p-value was 0.001, which suggests linear correlations between ACL tear and hyperextension of the knee joint (Table 5). 

**Table 5:** Statistical analysis of radiologically assessed hyperextension of the knee joint.

Radiological Parameters		Hyperextension (Affected Knee)	Hyperextension (Normal Knee)
Hyperextension (Affected knee)	Pearson Correlation	1	0.910
	Sig. (2-tailed)		0.001
	N	80	80
Hyperextension (Normal knee)	Pearson Correlation	0.910	1
	Sig. (2-tailed)	0.001	
	N	80	80

**Figure 6:**
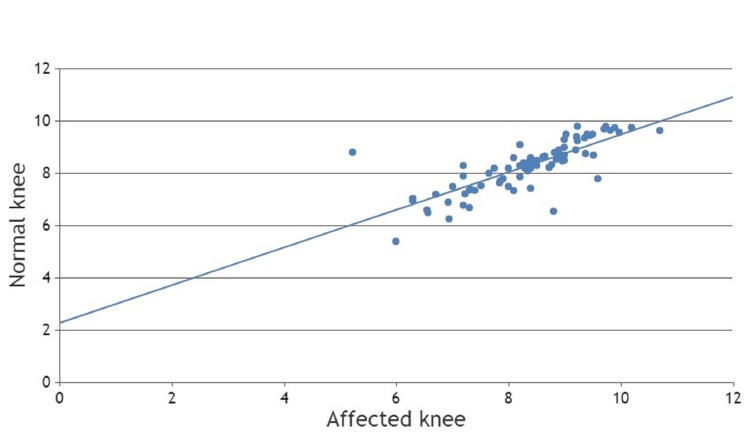
Scatter chart of the posterior tibial slope between the affected knee and the normal knee.

**Figure 7:**
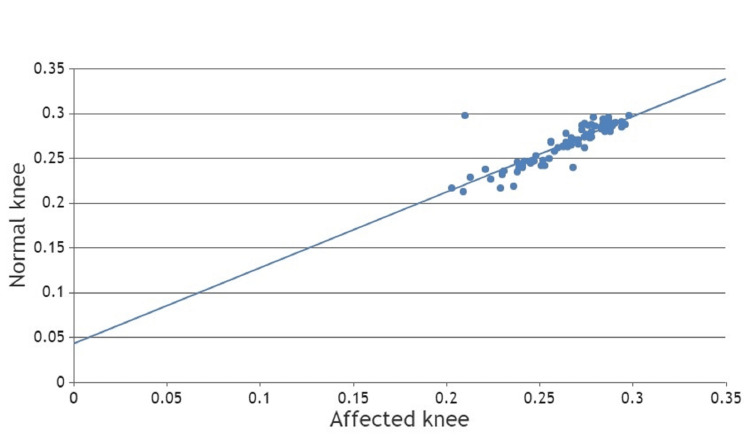
Scatter chart of notch width index between affected and normal knee.

We also measured the posterior tibial slope (Figure 6) and notch width index (Figure 7) of the ACL-deficient and contralateral uninjured knee joints, which were insignificant; the p-values were 0.844 and 0.547, respectively, which suggested that there was no significant difference in the posterior tibial slope and notch width index of the ACL-deficient knee and contralateral normal knee joint. We also found that there is no correlation between hyperextension of the knee joint and posterior tibial slope (Table 6). We also found that there is no correlation between hyperextension of the knee joint and the Notch width index (Table 7).

**Table 6:** Statistical analysis of radiologically assessed hyperextension and posterior tibial slope (TS).

Radiological Parameters		Tibial slope (Affected Knee)	Tibial slope (Normal Knee)
Hyperextension (Affected knee)	Pearson Correlation	-0.018	-0.043
	Sig. (2-tailed)	0.877	0.704
	N	80	80
Hyperextension (Normal knee)	Pearson Correlation	-0.094	-0.042
	Sig. (2-tailed)	0.405	0.714
	N	80	80

**Table 7:** Statistical analysis of radiologically assessed hyperextension and Notch width index (NWI).

Radiological Parameters		Notch Width Index (Affected knee)	Notch Width Index (Normal knee)
Hyperextension (Affected knee)	Pearson Correlation	-0.068	0.055
	Sig. (2-tailed)	0.547	0.626
	N	80	80
Hyperextension (Normal knee)	Pearson Correlation	-0.050	-0.006
	Sig. (2-tailed)	0.658	0.959
	N	80	80

## Discussion

In the present observational study, out of 10 females, four had ACL injuries because of involvement in sports activity, while six had injuries because of knee twisting due to road traffic accidents and while doing household activities. Out of 70 male patients, 28 had ACL injuries due to sports activity, while 42 male patients had ACL injuries due to road traffic accidents. Most ACL injuries are secondary to road traffic accidents in our study. This could be attributed to the frequent involvement of men in road traffic accidents and sports activities in this country as compared to other countries [12]. During the assessment of hyperextension in 80 ACL-deficient patients, 52 patients had generalized ligament laxity.

ACL plays a significant and vital role in the prevention of anterior translation of the tibia over the fixed femur. It also gives the sense of a stable knee joint through the proprioceptors in it. However, its theoretical role in the prevention of knee hyperextension or excessive knee hyperextension leading to ACL tears has not been well studied. To the best of our knowledge, no detailed prospective study analyzing its role and comparing it with the contralateral knee joint has been done so far.

In the present cross-sectional study, we took the opposite knee of the same patient as a control group to minimize the assessment bias. Clinically measured knee hyperextension angle may or may not be accurate and can also have inter-observer variation, so we additionally calculated roentgenogram-based knee hyperextension angle. The present study’s statistically analyzed data suggests a linear correlation between ACL deficiency and knee hyperextension and confirms the definite role of ACL in the prevention of knee hyperextension in a much more scientific manner as compared to previous studies.

Guimaraes et al. found that knee hyperextension greater than 5° is a significant risk factor for failure in ACL reconstruction with hamstring grafts. This suggests that patients with excessive knee hyperextension may have poorer outcomes following ACL reconstruction surgery, highlighting the importance of assessing and addressing knee hyperextension in preoperative planning and postoperative rehabilitation protocols for ACL reconstruction patients [13]. Saita et al. found that knee hyperextension and a smaller lateral condyle were correlated with greater quantified anterolateral rotatory instability in individuals with a complete ACL rupture [14]. Larson et al. revealed in their study that individuals with generalized hypermobility and knee hyperextension had worse outcomes after ACL reconstruction compared to those without these characteristics [15]. Fuss et al. elucidated the role of the cruciate ligaments, particularly the anterior cruciate ligament and posterior cruciate ligament, in limiting extreme ranges of motion in the knee joint [16].

In our study, we found that ACL injury causes hyperextension post-injury. Multiple studies have postulated the importance of assessing and addressing knee hyperextension, whether pre-existing or post-injury, and it plays an important role in surgical planning and also in rehabilitation.

The strengths of our study were its sample size, the use of the opposite normal knee as a control, which reduced the chance of bias, and the fact that we measured the hyperextension both clinically and radiologically. The limitations of our study were that we did not have a control group. We did not correlate the hyperextension with the severity of instability, and we did not measure the amount of hyperextension post-ACL reconstruction.

## Conclusions

Anterior cruciate ligament complete tear or deficiency produces knee hyperextension, which varies from patient to patient. Though the amount of hyperextension produced is mild (less than five degrees in most patients), it can cause a significant amount of knee instability. Hence, correction of knee hyperextension is crucial while performing ACL reconstruction.
